# Isolation and Characterization of a *Shewanella* Phage–Host System from the Gut of the Tunicate, *Ciona intestinalis*

**DOI:** 10.3390/v9030060

**Published:** 2017-03-22

**Authors:** Brittany Leigh, Charlotte Karrer, John P. Cannon, Mya Breitbart, Larry J. Dishaw

**Affiliations:** 1 College of Marine Science, University of South Florida, St. Petersburg, FL 33701, USA; bleigh@mail.usf.edu (B.L.); mya@usf.edu (M.B.); 2 Department of Pediatrics, University of South Florida, St. Petersburg, FL 33701, USA; ckarrer@mail.usf.edu (C.K.); jcannon@health.usf.edu (J.P.C.)

**Keywords:** *Shewanella*, bacteriophage, biofilm, extracellular DNA

## Abstract

Outnumbering all other biological entities on earth, bacteriophages (phages) play critical roles in structuring microbial communities through bacterial infection and subsequent lysis, as well as through horizontal gene transfer. While numerous studies have examined the effects of phages on free-living bacterial cells, much less is known regarding the role of phage infection in host-associated biofilms, which help to stabilize adherent microbial communities. Here we report the cultivation and characterization of a novel strain of *Shewanella fidelis* from the gut of the marine tunicate *Ciona intestinalis*, inducible prophages from the *S. fidelis* genome, and a strain-specific lytic phage recovered from surrounding seawater. In vitro biofilm assays demonstrated that lytic phage infection affects biofilm formation in a process likely influenced by the accumulation and integration of the extracellular DNA released during cell lysis, similar to the mechanism that has been previously shown for prophage induction.

## 1. Introduction

A significant proportion of microbes in the marine environment, including both bacteria and bacteriophages (i.e., phages), are associated with eukaryotic hosts, where they form stable symbiotic relationships. These symbiotic relationships are often specific and necessary in maintaining animal health via carefully orchestrated exchanges (i.e., homeostasis). Although phages are the most abundant biological entities in the natural world [1,2], little is known about their role in structuring and maintaining host-associated microbial communities, or how they influence bacteria within a biofilm, a lifestyle many aquatic bacteria exhibit. Even less is known about how perturbation of these microbial communities influences the eukaryotic host. Many animals maintain a “core” assemblage of bacteria (i.e., a core microbiome) that likely provides advantages to the host [3,4,5]. Some of these bacteria are consistently found within the same environments (e.g., animal intestines) and across diverse animal hosts, where they are presumed to serve distinct functions for either the animal host and/or the surrounding microbes. One such bacterial genus is *Shewanella* [6,7,8,9].

*Shewanella* species from a wide range of environments are known for their highly versatile metabolic capabilities that utilize diverse electron acceptors including nitrate, nitrite, thiosulfate, elemental sulfur, iron oxide and manganese oxide [10,11,12]. *Shewanella* species shuttle electrons across their membranes during anaerobic respiration, resulting in electrical activity within their biofilms and the transformation of insoluble compounds to bioavailable ones. Interestingly, biofilms with electrical activity have been documented to influence host cellular responses [13]. These bacteria make stable biofilms and because they can respire almost any compound, they likely represent important symbionts of animals as well. Despite extensive genomic rearrangements within *Shewanella* genomes [14], members of the genus retain a core set of metabolic genes that facilitate their survival in diverse environments [15], including the gut of a number of organisms [6,7,8,9]. *Shewanella putrefaciens*, which is closely related to *S. fidelis*, has shown promise as a probiotic for aquaculture [16], further emphasizing important roles for *Shewanella* in aquatic animal-microbe relationships.

To date, a number of *Shewanella* phages (both lytic and temperate) have been described from marine and freshwater environments [17,18,19,20,21], and in *Shewanella oneidensis*, prophages have also been implicated as vital for biofilm formation through excision-mediated lysis [21]. Stably integrated prophage-like elements are common within the genomes of most marine bacterial species [22], and prophages are also thought to be important among bacteria that colonize the gut mucosa of animals [23], often forming biofilms [24,25]. These biofilms are thought to serve as physical structures that can enhance pathogen defense by contributing to physical barriers, and through the production of diverse antimicrobials [26,27]. 

To begin to understand the role of phages in shaping the microbiome of sessile, filter-feeding marine invertebrates, we isolated and characterized a core member of the gut microbiome in the tunicate, *Ciona intestinalis* [4]. This novel strain of *Shewanella fidelis* (3313) was sequenced, and its inducible prophages and a strain-specific lytic phage (SFCi1, which was isolated separately from seawater) were characterized. Previously, it has been shown that spontaneous prophage induction can augment biofilms in some strains of bacteria [28,29,30,31]; we demonstrate here that infection of *S. fidelis* 3313 by lytic phage SFCi1 also enhances biofilm formation in vitro in a similar DNA-dependent manner. 

## 2. Materials and Methods

### 2.1. Bacterial Isolation from the Gut of Ciona Intestinalis

*Ciona intestinalis* specimens were collected from Mission Bay in San Diego (M-REP Animal Collection Services, San Diego, CA, USA) during the Spring of 2014. Animals were cleared for 48 h in seawater filtered through a 0.22 μm pore size filter (Millipore Sterivex, Merck, Darmstadt, Germany) (with water changes every several hours), before the entire gut (stomach, midgut, hindgut) of five animals was dissected and homogenized using a dounce homogenizer. The gut homogenate was filtered through a 0.45 μm pore size filter (Millipore Sterivex, Merck) to remove host tissue, and the bacteria were pelleted by centrifugation at 12,500 *xg* for 10 min and washed three times through resuspension and centrifugation in 1 mL of sterile (filtered through a 0.22 μm pore size filter and autoclaved) artificial seawater (Instant Ocean AS9519, Marine Depot, Garden Grove, CA, USA). Serial dilutions of the bacterial homogenate were plated on marine agar (MA) 2216 (Becton Dickinson Company, Franklin Lakes, NJ, USA). Colonies displaying distinct phenotypes were randomly chosen, purified by streaking, and grown separately in the corresponding liquid broth (marine broth (MB) 2216, pH 7.6) at 20 °C; subsequently, a 20% glycerol stock was made for each isolate and stored at −80 °C. DNA was isolated using the PowerSoil DNA Kit (MoBio Laboratories, Carlsbad, CA, USA) and the 16S rRNA gene amplified using universal primers 27F and 1492R [32] (polymerase chain reaction (PCR) conditions: denature at 95 °C for 5 min, cycle 35 times through 94 °C for 30 s, 56 °C for 30 s, 72 °C for 1 min 30 s, and end with a final extension at 72 °C for 10 min), sequenced via the Sanger platform and identified using BLAST against the NCBI non-redundant database [33]. 

### 2.2. Phage Isolation, Propagation, and Purification for Transmission Electron Microscopy

*S. fidelis* 3313 recovered from the *Ciona* gut homogenate was screened for lytic phages via standard plaque assays using seawater from which the animals were shipped (i.e., bag water) filtered through a 0.22 μm pore size filter. Approximately 500 mL of the filtered seawater was concentrated using Amicon Ultra-15 concentration units (molecular weight cut-off (MWCO) 100 kDa; EMD (Merck Millipore, Darmstadt, Germany) by centrifugation to a final volume of ~15 mL. Lytic phages were isolated with the double agar method (0.5% low-melt top agar) [34] using the prepared seawater concentrate and the bacterial host grown to log phase (OD_600_ = 0.25) in MB. Each plaque was then cored, plaque-purified three times and resuspended in 500 μL of sterile modified sodium magnesium (MSM) buffer (450 mM NaCl, 102 mM MgSO_4_, 50 mM Tris Base, pH 8). The purified phage was propagated on *S. fidelis* 3313 lawns on MA at room temperature. The resulting lysate was filtered through a 0.22 μm pore size filter and stored in MSM buffer at 4 °C.

To estimate phage–host growth dynamics including the latent period and burst size, a one-step infection curve was performed according to Hyman and Abedon [35], with slight modifications. Latent period is defined as the period between the adsorption time and the initial phage lysis of the bacterial culture, prior to any significant rise in phage particles [35]. For this procedure, a 10 min adsorption step at a multiplicity of infection (MOI) of 1 was followed by centrifugation at 13,000× *g* for 30 s to pellet the bacteria with adsorbed phages. The pellet was then resuspended in 1 mL of sterile MB. Triplicate samples were taken at 10 min intervals for up to 2 h and directly plated using the double agar method to determine phage titer. Additionally, burst size was measured as the ratio of final phage particles to the number of bacterial cells at the onset of phage exposure.

A portion of the lysate was further purified via cesium chloride (CsCl) gradient ultracentrifugation [36] for morphological analysis by transmission electron microscopy (TEM) using an Hitachi 7100 (Hitachi Ltd., Tokyo, Japan). The purified virus particles were prepared for imaging on a formvar grid (Electron Microscopy Sciences, Hatfield, PA, USA) using a negative stain with 2% uranyl acetate, as described previously [37]. Images were captured using an Orius SC600 bottom mount camera (Gatan Inc., Pleasanton, CA, USA) at 100 kV. A separate aliquot of this purified viral suspension was reserved for DNA extraction using the QIAmp MinElute Virus Spin Kit (Qiagen Inc., Valencia, CA, USA) and for sequencing, as described below.

### 2.3. DNA Extraction, Sequencing and Analysis

*Shewanella fidelis* 3313 was cultured in MB overnight at 20 °C with shaking at 90 RPM, and its lytic phage was propagated and purified as described above. Bacterial DNA was extracted using the PowerSoil DNA Kit (MoBio Laboratories, Carlsbad, CA, USA) as described above. All viral DNA was amplified using a GenomiPhi V2 DNA amplification kit (GE Healthcare Life Sciences, Pittsburgh, PA, USA) to generate adequate template for sequencing (~1 μg). To minimize bias introduced by the amplification process, three identical reactions were prepared and pooled.

Bacterial, phage and prophage DNA were sequenced with the Illumina MiSeq platform generating mate-pair (2 × 250) libraries (Operon, Eurofins MWG Operon LLC, Huntsville, AL, USA). The NuGen UltraLow DNA kit was used (Eurofins, Louisville, KY, USA) to prepare libraries, and DNA quality was assessed on a BioAnalyzer 2100 (Agilent Technologies, Santa Clara, CA, USA), with size selection (400–600 bp) conducted to remove outlier DNA fragments after sonication. The resulting mate-pair reads were assembled using approaches described in Deng et al. [38], first by a de Bruijn graph assembler (Velvet de novo assembler with a k-mer of 35 (phage) and 27 (bacteria) [39]), followed by the default consensus algorithm in Geneious 8.1.7 (Biomatters Ltd, Auckland, New Zealand) [40]. Viral genome open reading frames (ORFs) were identified using Glimmer3 [41] through Geneious 8.1.7; annotations were improved with the BLASTX algorithm against non-redundant protein databases in GenBank, Protein Data Bank (PDB), SwissProt, Protein Information Resource (PIR) and Protein Research Foundation (PRF) via Geneious 8.1.7. All resulting bacterial contigs were uploaded to the Rapid Annotation using Subsystem Technology (RAST) server [42,43] under sample ID mgs422948, with full annotations based on the SEED Database. All contigs passed Metagenomics (MG)-RAST quality control and all predicted proteins were annotated. The complete 16S rRNA gene of *S. fidelis* 3313 was compared via PHYML maximum likelihood trees to other described *Shewanella* species in the GenBank database (Figure S1). The closest species was determined to be *Shewanella fidelis* (ATCC BAA-318; GenBank ID: 17801). All assembled contigs greater than 1500 bp (a total of 24) were compared to this nearest neighbor to determine average nucleotide identity (ANI) of the entire genome using the ANI calculator [44]. Genomes with ANI values above 95% are considered to belong to the same species [45].

### 2.4. Prophage Induction and Identification

Prophage regions were identified and annotated by screening all bacterial contigs using the VirSorter pipeline [46]. To determine if *S. fidelis* 3313 possessed any inducible prophages, mitomycin C was introduced to an early log-phase culture (OD_600_ of 0.025) at a final concentration of 1 μg/mL and incubated for 24 h [47]. The resulting culture, along with an untreated control culture, was then stained with SYBR Gold nucleic acid stain and induced phage particles were enumerated using epifluorescence microscopy, as described previously in Patel et al. [48]. Induced phage particles were then CsCl-purified and treated with 2U of DNase I Turbo (Invitrogen, Carlsbad, CA, USA), before DNA was extracted using the QIAmp MinElute Virus Spin Kit (Qiagen Inc.). Mate-pair (2 × 250) libraries were produced using the NuGen UltraLow DNA kit, quality controlled with the BioAnalyzer as previously stated (Operon, Eurofins MWG Operon LLC), and sequenced on the Illumina MiSeq platform. Reads were then mapped back to the assembled *S. fidelis* 3313 genome utilizing the Geneious 8.1.7 software, with default parameters to determine which of the predicted prophages were induced. Additionally, TEM analysis of the CsCl-purified induced prophage fraction was performed as described above.

### 2.5. Biofilm Assays

Single colonies of *S. fidelis* 3313 were grown in MB at 20 °C overnight with shaking at 90 RPM. Concentration was estimated by optical density at OD_600_ based on previously-calibrated growth curves and colony forming units (data not shown). For biofilm assays, stationary cultures were diluted to a final concentration of 10^6^ cells mL^−1^ (early log phase OD_600_ of 0.025), and phages were added at 10^6^ plaque forming units (PFUs) mL^−1^ immediately before plating. All bacterial treatments and controls were plated on 12-well plates (Thermo Scientific, Waltham, MA, USA) in triplicate; bacteria were also plated in duplicate on 35 mm glass bottom dishes (No. 1.5, uncoated; MatTek Corporation, Ashland, MA, USA) for extracellular DNA detection using the TOTO-1 Iodide 514/533 stain (Molecular Probes, Invitrogen) and counterstained for live cells using SYTO60 red (Molecular Probes, Invitrogen) [49]. Additionally, purified salmon sperm DNA (Invitrogen) was added to separate cultures at a final volume of 300 ng mL^−1^. All stationary culture dishes were incubated at 20 °C for up to two days to allow biofilm formation, before excess liquid and planktonic bacteria were removed by gentle pipetting. To quantify biofilm formation, culture dishes were allowed to dry, and then subsequently stained with a 0.1% crystal violet solution for 10 min, as per Merritt et al. [50]. Crystal violet was removed by decanting, and the dishes were washed twice with distilled water to remove excess stain and then allowed to dry completely. The dried crystal violet was resuspended in 30% acetic acid for ~10 min, and the OD_590_ was determined for each culture dish [50]. At each time point, biofilms in the 12-well dishes and one of the MatTek dishes at 24 h (when the difference was the most drastic) were treated with 2U of DNase I Turbo (Invitrogen), for 10 min at 37 °C. Both dishes for fluorescent microscopy staining were then washed once with 1× PBS before TOTO-1 staining for 10 min. Excess dye was then removed and the biofilm washed twice in 1× PBS before counterstaining with SYTO60 for 10 min. Excess dye was removed, washed once in 1× PBS and held in 1× PBS for imaging of 10 random fields using the Leica Application Suite (Leica Microsystems, Wetzlar, Germany) and the Metamorph version 7.5 software (Molecular Devices, LLC, Sunnyvale, CA, USA), using consistent exposure, aperture, and magnification settings. Images were exported as TIF files from Metamorph (Molecular Devices) and imported into ImageJ 1.48v [51]. Without any additional enhancements, the channels were separated, thresholds permanently set, signal intensity was averaged and standard deviations determined over the area selected.

## 3. Results

### 3.1. Bacterial Cultivation and Genome Sequencing

The 16S rRNA gene of *S. fidelis* 3313 is identical to the V3-V4 region of a core operational taxonomic unit (OTU) described previously from the *Ciona* gut [4]; members of the core microbiome likely are of functional relevance within the host, perhaps provisioning nutrients or essential elements, or contributing to the establishment of gut-associated bacterial communities. Next-generation sequencing of the *S. fidelis* 3313 genome resulted in 5,940,000 reads which assembled into 129 scaffolds (N_50_ = 937,903), with an average coverage of 527× and a mean guanine-cytosine (GC) content of 43.3%. Collectively, these scaffolds contained 3,536 predicted ORFs, all of which could be annotated through at least one of the four MG-RAST protein databases [42,43]. Pairwise sequence identity comparisons and nearest neighbor analyses suggest that isolate 3313 from the *Ciona* gut is related most closely to a partially-sequenced *S. fidelis* species previously isolated from sediments and seawater in the South China Sea [52]. The near-full-length 16S rRNA gene of the *S. fidelis* 3313 isolate is 99% identical to this previously-described *S. fidelis* (Figure S1), and was deposited separately into GenBank (ID: KY696838); however, the assembled genomic contigs reveal an average overall nucleotide identity of 97.35% (Figure S2), making isolate 3313 a novel strain of *Shewanella fidelis* [45,53].

### 3.2. Prophage Induction and Genome Sequencing

**Figure 1:**
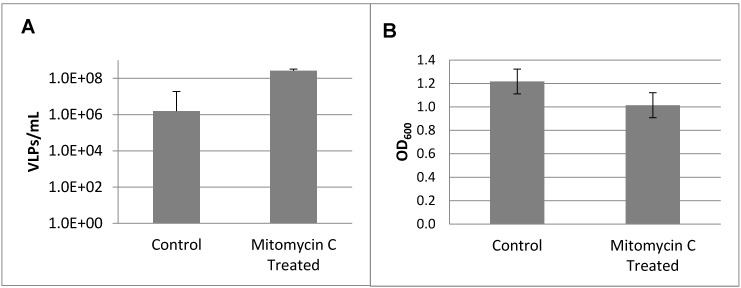
(**A**) Viral-like particles (VLPs) enumerated via epifluorescence microscopy after mitomycin C induction of *Shewanella fidelis* 3313. (**B**) Bacterial turbidity measured at OD_600_. All measurements were recorded at 24 h after treatment.

VirSorter screening of the *S. fidelis* 3313 assembled genome revealed the presence of at least three genetic loci with sequence similarities to previously-described prophages. To determine if *S. fidelis* 3313 possessed inducible prophages, cultures in early log phase (OD_600_ = 0.025) were treated overnight with mitomycin C, a commonly-used mutagen for prophage induction; the resulting virus-like particles (VLPs) were enumerated via epifluorescence microscopy (Figure 1). Mitomycin C induction resulted in an increase in VLPs (Figure 1A), concurrent with a decrease in culture turbidity as measured by optical density (Figure 1B).

**Figure 2:**
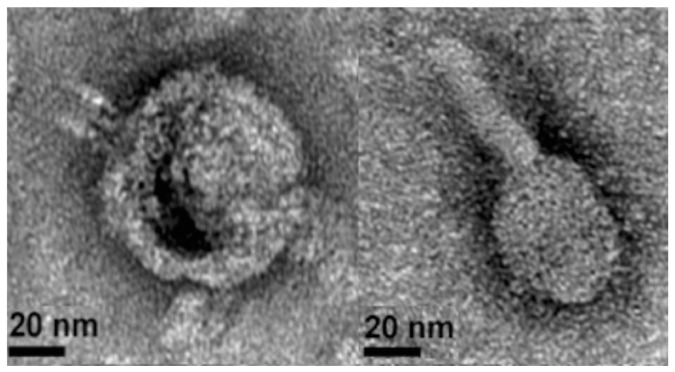
Representative transmission electron microscopy (TEM) images of mitomycin C-induced phages recovered from the supernatant of *S. fidelis* 3313.

Imaging of the same supernatant after cesium chloride (CsCl)-purification by transmission electron microscopy (TEM) confirmed two morphologies of intact phage particles (Figure 2). The DNA of these purified phage particles was then sequenced and mapped to the *S. fidelis* 3313 genome, verifying that they represent two intact and active prophage elements. The first prophage, detected in contig 6, spans 19,321 bp and is predicted to encode 31 genes. This prophage, hereby referred to as SFPat, encodes mostly hypothetical proteins. The second prophage, hereby referred to SFMu1, was detected in contig 7, spans 45,796 bp, and is predicted to encode 57 genes. Phage SFMu1 possesses several Mu-like gene elements described previously in a number of Gram-negative bacteria, including *Shewanella oneidensis* [54]. Annotations of predicted ORFs corresponding to each of these elements are outlined in Supplementary Materials Tables S1–S2.

### 3.3. Lytic Phage Isolation and Genome Sequencing

**Figure 3:**
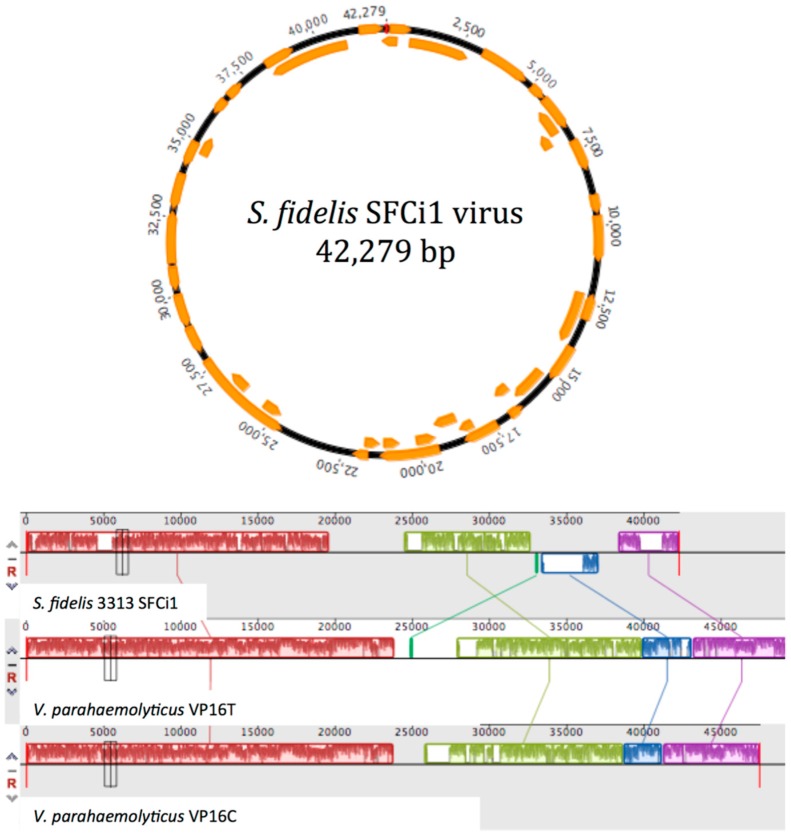
(**A**) Circular genome of lytic phage SFCi1 depicts 40 open reading frames (ORFs). (**B**) Mauve alignment of the SFCi1 genome against two most closely related phage genomes, VP16C and VP16T. Colored boxes indicate sequence blocks with shared sequence identity; regions lacking sequence homology are indicated in white.

To identify phages capable of lytic infection of *S. fidelis* 3313, water from the collection site (also used in shipping live animals) was sequentially filtered, concentrated, and screened by standard plaque assays. A candidate lytic phage was identified, amplified, purified, and sequenced. The *S. fidelis* 3313 lytic phage, named SFCi1, has a circular, double-stranded DNA genome consisting of 42,279 bp (38,089× genome coverage), and a GC content of 59.1% (Figure 3).

**Figure 4:**
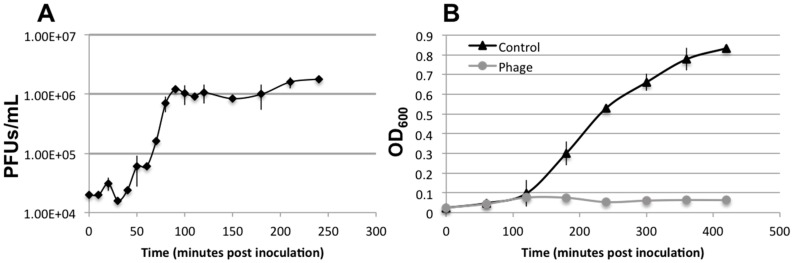
One-step growth curve of lytic phage SFCi1 with its host, *S. fidelis* 3313, as determined by plaque forming units (PFUs) and bacterial turbidity measurements (OD_600_).

The assembled genome is predicted to possess 40 ORFs, summarized in Supplementary Materials Table S3. The infection curve generated by the SFCi1 phage revealed a decline in optical density of the *S. fidelis* 3313 culture within one-hour post-infection (approximate latent period), with complete culture lysis by 4 h (Figure 4). Additionally, the average burst size was calculated to be 62.

**Figure 5:**
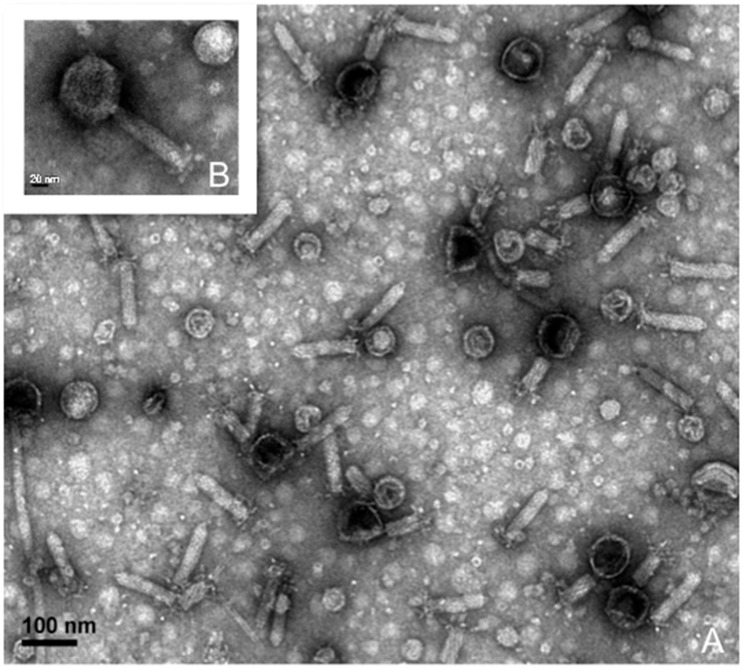
(**A**) TEM image of a pure culture of lytic phage, SFCi1, indicates that it is a myophage. (**B**) Magnified image of a single viral particle outlining the icosahedral head and details of the contractile tail and baseplate.

Morphological analysis via TEM suggests that SFCi1 belongs to the *Myoviridae* family [55], with a capsid diameter of approximately 70 nm (StDev: 7.4 nm), and a tail length of approximately 120 nm (StDev: 5.5 nm) (Figure 5). Members of this family possess a contractile tail through which DNA is inserted into the bacterial cell after degradation of surface structures by lysozyme, an enzyme also encoded in the SFCi1 genome. Phage SFCi1 shares the greatest sequence similarity with two siphophages (VP16C and VP16T) isolated from San Diego that infect *Vibrio parahaemolyticus* [56]. However, SFCi1 is unable to infect the *Vibrio parahaemolyticus* host of VP16C/T (data not shown). Figure 3, which depicts the regions of nucleotide identity between the three phage genomes via Mauve alignment [57], reveals substantial syntenic regions. It is notable that the genes encoding the tail of SFCi1 are more similar to that of *Vibrio* phage H188, a recently discovered myophage from the Yellow Sea [58]. The tail proteins characteristic of myophages generally allow for a broader host range than that seen for siphophages [59]. The SFCi1 phage genome was also placed onto the phage proteomic tree [60], which approximated the closest viral relatives as VP16T/C (Figure S3).

### 3.4. Similarity to Vibrio Phages

Because phages utilize host machinery for protein translation during replication, their codon usage tends to evolve towards that of the host genome [61]. The codon adaptation index (CAI), which is expected to increase upon phage–host coevolution, was higher between *S. fidelis* 3313 and the three phages examined (SFCi1 (0.753), VP16C (0.747), and VP16T (0.758)), than between *Vibrio parahaemolyticus* (RIMD 2210633) and those phages (SFCi1 (0.624), VP16C (0.607), and VP16T (0.604)). No tRNAs were detected in the SFCi1 phage genome that could independently influence the codon usage bias [62].

### 3.5. Biofilm Development

**Figure 6:**
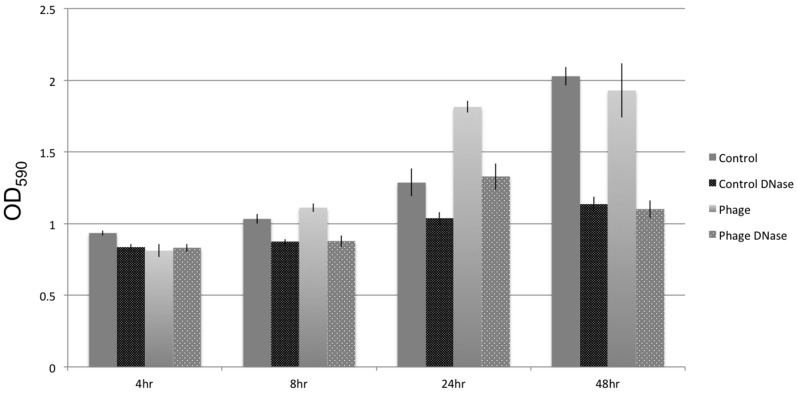
Development of a biofilm by *S. fidelis* over 48 h in stationary culture, as measured by crystal violet staining of the biofilm, in the presence (Phage) and absence (Control) of lytic phage SFCi1 and/or DNase I treatment.

In vitro, the addition of lytic phage SFCi1 into pure static cultures of *S. fidelis* 3313 caused an increase in biofilm formation, beginning at 8 h post addition, and led to a more robust biofilm by 24 h, as detected by crystal violet staining of adherent biofilm structures (Figure 6).

**Figure 7:**
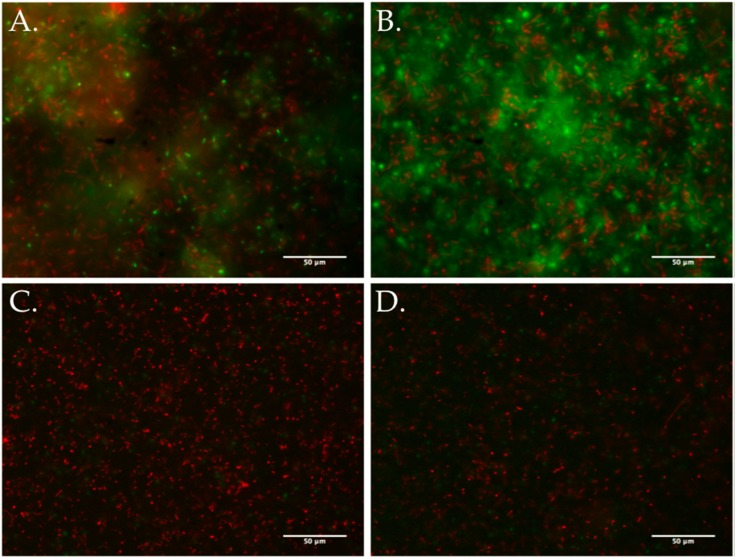
TOTO-1 Iodide 514/533 stain and SYTO60 red counterstain reveals extracellular DNA as a major component of the stationary culture biofilm. (**A**) Untreated *S. fidelis* 3313 control culture (30.23% ± 5.1% pixel area coverage); (**B**) *S. fidelis* 3313 exposed to SFCi1 lytic phage (80.96% ± 7.9% area); (**C**) control culture treated with DNase I (0.754% ± 0.01% area); and (**D**) lytic-phage treated culture co-treated with DNase I (0.772% ± 0.017% area).

Similar effects on biofilm formation could be achieved with the inclusion of foreign DNA, such as salmon sperm DNA, into the cultures (data not shown). When DNase I was added to static cultures, whether or not exposed to lytic phages, biofilm density decreased. This suggests extracellular DNA is an important structural component of the *S. fidelis* 3313 biofilms, seemingly as a result of cell lysis. The extracellular DNA was quantified by measuring fluorescent signal (TOTO-1 Iodide 514/533 stain, Molecular Probes), and determining the percent area coverage represented by the labeled extracellular DNA signal. Fluorescent microscopy images depicted an increase in free extracellular DNA (80.96% ± 7.9% pixel area coverage) within the biofilm-rich cultures that include phage SFCi1 (Figure 7). Control cultures also revealed the presence of extracellular DNA (30.23% ± 5.1% pixel area coverage), suggesting that lysis by spontaneous prophage induction as shown in controls in Figure 1) may play a role in natural biofilm formation by this strain. Treatment with DNase I resulted in the depletion of extracellular DNA from both the control (0.754% ± 0.01% area) and phage SFCi1 (0.772% ± 0.017% area) cultures. However, DNase I alone was not capable of completely eliminating detectable biofilms, suggesting that extracellular DNA is not the only requirement for biofilm formation.

### 3.6. GenBank Accession Numbers

*Shewanella fidelis* 3313 SFCi1 virus was submitted to GenBank under the Accession ID number KX196154. The *Shewanella fidelis* 3313 16S rDNA gene has been submitted to GenBank under the Accession ID number KY696838. The *Shewanella fidelis* 3313 draft genome is available in MG-RAST under sample ID mgs422948.

## 4. Discussion

Despite being surrounded by abundant and diverse microbes, filter-feeding sessile aquatic invertebrates maintain stable and often species-specific resident microbial communities (i.e., core microbiomes) [3,4,5]. A stable microbiome can contribute gene products of functional relevance, influencing metabolic pathways and nutrient acquisition [63]. *Ciona intestinalis* is a marine protochordate with a fully sequenced genome that is being developed for gut microbiome studies utilizing germ-free mariculture [64]; recent evidence also indicates the presence of a core microbiome [4]. Culturing members of the *Ciona* gut microbiome, along with corresponding lytic phages, is a first step in designing experimental approaches to study the processes governing bacterial colonization of mucosal surfaces. Mono-association studies in *Ciona* (e.g., with *S. fidelis* 3313) could provide key insights into host-bacterial interactions at the onset of colonization in naïve tissue surfaces. Furthermore, investigations of mixed community colonization could reveal patterns of succession that are influenced by the activity of phages, along with other external and host-derived factors [65,66,67].

The establishment and maintenance of homeostasis between a host and its gut microbiome is an exceedingly complex, multifaceted process, likely to be influenced by many parameters, including external factors (e.g., nutrient availability, other microbes and microbial products), viruses that infect these microbes (i.e., phages), and host factors (e.g., mucus, immunity). The abundance, ubiquity, and high genetic diversity of phages, together with their roles in shaping bacterial communities and their influences on horizontal gene transfer [2,68,69,70], suggest that phages are highly influential members of complex microbial communities [71]. Filter-feeding invertebrates come into contact with a continuous assortment of microbes and free phages, which have the potential to profoundly alter community dynamics in established microbiomes. In addition to the free phages in seawater (~10^7^ phages per milliliter [2]), most marine bacterial species have been documented to possess stable and active prophages (i.e., phages integrated into bacterial genomes) [22]. While an increasing number of studies are examining the diversity and variability of phages within microbiomes [72,73,74,75], little is known about how these phages influence microbiomes and their role(s) in homeostasis.

In the *S. fidelis* 3313 draft genome assembly, a total of three prophage regions were identified by VirSorter. Activity of two of these prophages was confirmed experimentally through the identification and sequencing of intact viral particles after induction of live cultures with the mutagen mitomycin C. Prophage SFPat (from contig 6) consists largely of hypothetical proteins. However, prophage SFMu1 (from contig 7) possesses genes more similar to those in public databases, facilitating annotation. For example, the Mu-like phages replicate via DNA transposition, and have evolved genes such as a *cis*-acting transposition enhancer and a centrally-located strong gyrase binding site [76], genes that also are present in SFMu1.

From the animal’s surrounding seawater, this study also identified a lytic phage, SFCi1, which infects *S. fidelis* 3313. Placement of the SFCi1 genome on the phage proteomic tree identified the *Vibrio* phages VP16T and VP16C as the closest relatives. Genome comparisons of SFCi1 to all other known *Shewanella* phages indicated little to no similarity (data not shown), and with less than 95% ANI to any other phage, SFCi1 represents a new phage species. Codon usage assessments suggest that SFCi1 may have infected *Shewanella* long before it encountered and/or infected *Vibrio parahaemolyticus*, which is consistent with previous observations that VP16C/T only recently infected *V. parahaemolyticus* [56]. Both *Shewanella* and *Vibrio* species are abundant in the marine water column, and horizontal gene transfer between the two bacterial families has been described [77]. Genetic exchange between the VP16C/T phages and phage SFCi1 could represent similar examples.

Previous studies have suggested that phages may influences bacterial competition in the environment [78], a phenomenon that likely also affects gut ecosystem dynamics. Activated prophages are known to influence biofilms by facilitating the transient liberation of extracellular DNA, which can then become a component of the biofilm matrix [21,31]. Biofilms can shelter microbial inhabitants from both physical and mechanical stressors, and consist mostly of microbially-derived polysaccharides, nucleic acids and lipids. Extracellular DNA has been shown to be an important component of biofilms in several species [79]. While the extracellular DNA is often composed of chromosomal DNA from the biofilm-producing bacteria [80], foreign extracellular DNA can similarly influence the growth and maturation of biofilms, suggesting that the origin of the DNA is less important than the structural support it lends [81]. Several sources of extracellular DNA within biofilms have been identified, including release in response to quorum sensing [82,83,84], autolysis [85], and prophage induction [21,30,86]. However, additional methods of extracellular DNA release by external factors such as lytic phages and host products (e.g., immune molecules, mucus) have yet to be elucidated in the context of biofilm formation.

Previously, *Vibrio anguillarum* phages have been shown to have different influences on biofilm formation over long-term cultures, due mostly to varied microcolony morphologies with one strain having flat single layers (BA35), and the other forming complex 3D structures (PF430-3) [87]. This structural distinction resulted in differential penetrability of the biofilm structure by phages during the initial stages of biofilm formation, and ultimately impeded the formation of the BA35 single layer biofilm compared to the control. Phage infection of BA35 resulted in a large percentage of resistant mutants (~70% of isolated cells); however, PF430-3 formed more complex 3D structures in the presence of its lytic phage, resulting in fewer resistant mutants. This result suggested that aggregation within these structures could be reducing phage adsorption [87]. The phage particles appeared to be trapped within the aggregate matrix, a complex consortia of extracellular polymeric substances of unknown composition that seemingly provided physical protection against phage infection; the mechanism for this differential aggregation phenotype has yet to be elucidated.

Lytic phage infection has also been shown to influence biofilm formation in *Pseudomonas aeruginosa*, *Salmonella enterica* and *Staphylococcus aureus*, with different outcomes in each bacteria-phage system; *S. aureus* was the only system where biofilm formation was enhanced [88], with *S. aureus* thought to transition to the biofilm phenotype as a means of physiological protection from phage infection. Enhanced formation of biofilms took place at early time points, with treated cultures returning to control levels over time, likely as a result of early biofilm dispersal. Although not investigated, the authors suggested that extracellular DNA released by lytic phage infection likely played a role in biofilm development [88].

The current study adds to this growing body of evidence regarding phage influence on biofilm formation, by demonstrating that lytic phage SFCi1 infection can similarly enhance biofilm formation in vitro by *S. fidelis* 3313. Fluorescence imaging shows that extracellular DNA is much more prominent in SFCi1 phage-exposed, biofilm-rich, cultures of *S. fidelis* 3313. The presence of some extracellular DNA in control cultures is consistent with spontaneous prophage induction. Treatment of phage-infected cultures with DNase I reduces biofilms, indicating that extracellular DNA is likely a major structural component of these biofilms. As seen for prophage induction, lytic phage dynamics can influence biofilm formation through the release of extracellular DNA. Additional mechanisms of biofilm enhancement have been described and include the generation of phage resistant mutants [89], quorum sensing [90], and the “wall effect”, whereby two subpopulations of the same strain co-exist with one protecting the other [91]. Whether any or all of these mechanisms are also involved in the phage-related biofilm increase described in *S. fidelis* 3313 remains to be demonstrated.

Among mucus-rich tissues, it has also been suggested that lytic phages can directly associate with mucus, a process that appears to influence selection of microbiota and/or help protect against certain pathogens [92]. However, the role of these phages at the gut mucus interface in vivo, and the ability of phages to infiltrate established biofilms and modify community structure, is not yet clear. Biofilms derived from distinct bacterial species, and sometimes composed from mixed communities, can demonstrate unique physical and chemical compositions of exopolysaccharides that exert distinct influences on phages [24,93]. Once phages become integrated into biofilms, some can be rapidly incorporated into the bacterial genome as prophages and lie dormant, making the bacterium a reservoir of future phage particles [94]. A variety of effectors can influence the induction of prophages. For example, viral excision from bacterial genomes in response to environmental stress has been implicated in biofilm restructuring and dispersal [31,86,95]. Since a large number of bacteria within the gut contain prophages, their induction likely imposes a significant restructuring of the associated microbial communities in response to host and environmental factors. However, natural triggers for prophage induction among members of mucus-associated microbiomes remain to be defined.

Phage manipulation of host mucosal epithelium-associated biofilms contributes to an already complex relationship between the host and its associated microbiome [96,97]. Characterizing members of the gut microbial community and their potential interactions is essential to understanding their influence on host health, and the establishment and maintenance of homeostasis. Deciphering these complex interactions is aided by carefully-designed in vitro and in vivo studies that enable careful control and observation of the dialogue between host immune factors and various members of the microbiome (including phages). Because bacteria in a biofilm often are recalcitrant to interventional therapies, dissection of the processes modulating the development and dispersal of biofilms in vivo within a natural host may aid in the development of new therapeutic approaches.

## Supplementary Materials

The following are available online at www.mdpi.com/1999-4915/9/3/60/s1, Figure S1: A PHYML-based tree (100 bootstrap replicates; 70% stringency) of *Shewanella* sp. 16S rRNA gene comparisons. Figure S2: Average nucleotide identity (ANI) comparison between the *S. fidelis* ATCC genome (GI:655363240) and *S. fidelis* 3313 as determined by the ANI Calculator (http://enve-omics.ce.gatech.edu/ani/); Figure S3: *S. fidelis* SFCi1 phage placement on phage proteomic tree. Table S1: ORF table for SFPat prophage; Table S2: ORF table for SFMu1 prophage; Table S3: ORF table for SFCi1 lytic phage.
