# Supplementary figures and images for: Isolation and Characterization of a Shewanella Phage–Host System from the Gut of the Tunicate, Ciona intestinalis
Source: Viruses. 2017 Mar 22;9(3):60. doi: 10.3390/v9030060 (PMC5371815; doi:10.3390/v9030060)

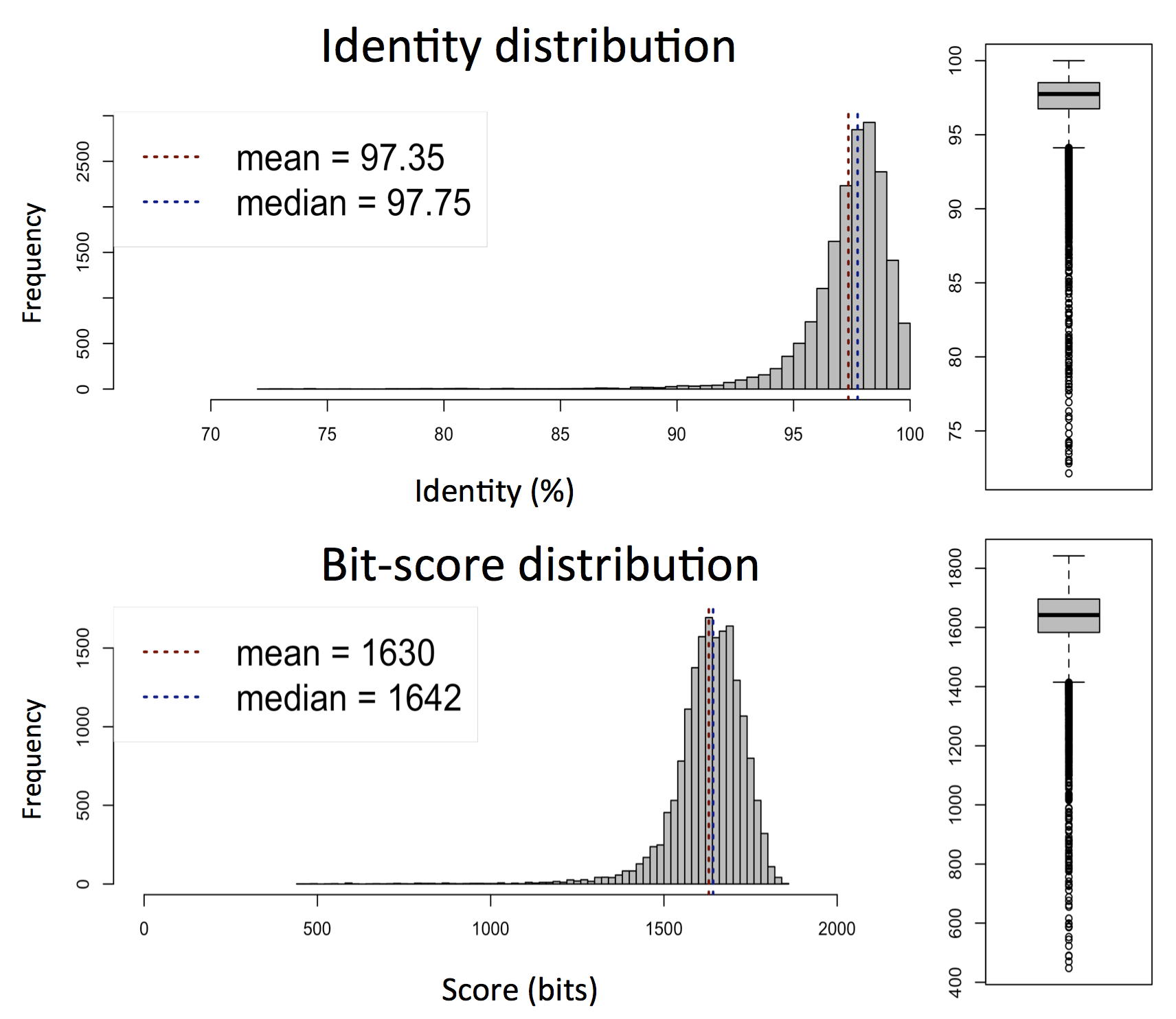

Supplement: Supplementary file 1 [file viruses-09-00060-s001.zip › FigS1.tiff]

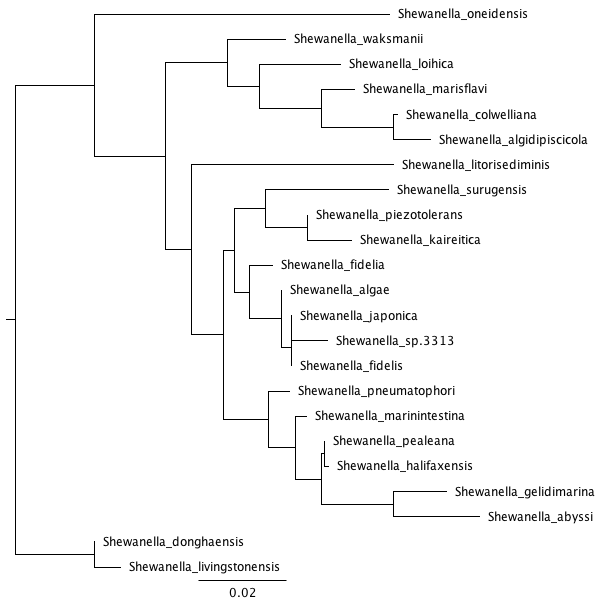

Supplement: Supplementary file 1 [file viruses-09-00060-s001.zip › FigS2_Shewanella16S_Tree.tif]

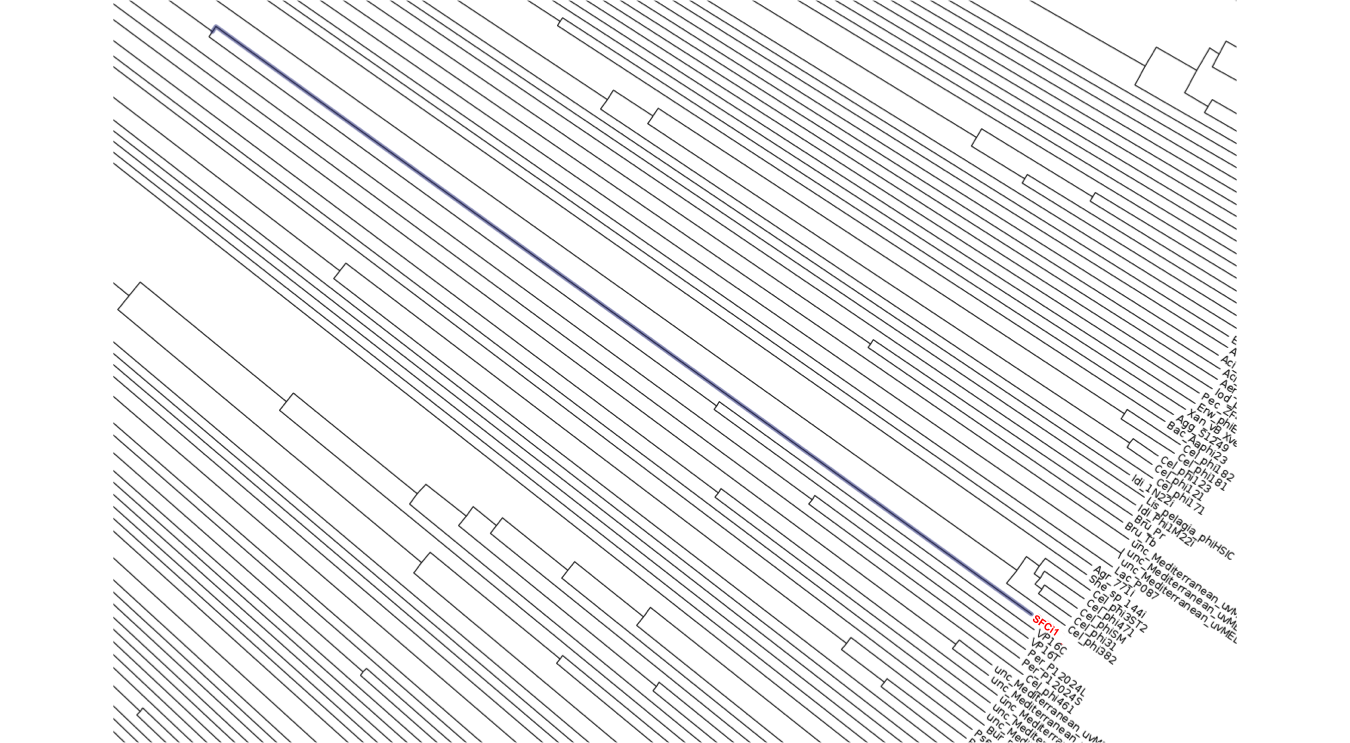

Supplement: Supplementary file 1 [file viruses-09-00060-s001.zip › FigS3_SFCi1_Tree.tif]
